# HPLC Analysis and Skin Whitening Effects of Umbelliprenin-containing Extracts of *Anethum Graveolens*, *Pimpinella Anisum*, and *Ferulago Campestris*

**DOI:** 10.3390/molecules24030501

**Published:** 2019-01-30

**Authors:** Vito Alessandro Taddeo, Francesco Epifano, Francesca Preziuso, Serena Fiorito, Nicolas Caron, Arnaud Rives, Philippe de Medina, Marc Poirot, Sandrine Silvente-Poirot, Salvatore Genovese

**Affiliations:** 1 Dipartimento di Farmacia, Università “G. d’Annunzio” of Chieti-Pescara, Via dei Vestini 31, 66100 Chieti Scalo (CH), Italy; vito.taddeo@unich.it (V.A.T.); francesca.preziuso@unich.it (F.P.); serena.fiorito@unich.it (S.F.); s.genovese@unich.it (S.G.); 2 Société de Biotechnologie médicale, Affichem S.A., 9 Rue Saint Joseph, 31400 Toulouse, France; n.caron@affichem.com (N.C.); a.rives@affichem.com (A.R.); 3 Cholesterol Metabolism and Therapeutic Innovations, Cancer Research Center of Toulouse (CRCT), UMR 1037, INSERM-Université de Toulouse, 31400 Toulouse, France; phildemedina@yahoo.fr (P.d.M.); marc.poirot@inserm.fr (M.P.); sandrine.poirot@inserm.fr (S.S.-P.)

**Keywords:** anise, apiaceae, dill, field ferula, melanin, melanogenesis, oxyprenylated coumarins, skin whitening effect, umbelliprenin

## Abstract

Umbelliprenin has recently been shown to have great potential as a skin whitening agent. Wishing to investigate the same effect in plant species known to biosynthesize this coumarin, three plants belonging to the Apiaceae family, namely *Anethum graveolens* L. (dill), *Pimpinella anisum* L. (anise), and *Ferulago campestris* (Besser) Grecescu (field ferula) were screened by HPLC analysis for their respective content of umbelliprenin in extracts obtained with different solvent mixtures and by maceration and ultrasound-assisted processes. EtOH was shown to be the best solvent, providing umbelliprenin yields ranging from 1.7% to 14.4% (with respect to the total amount of extract obtained). Extracts with the highest content of this farnesyloxycoumarin were then assayed as modulators of melanogenesis in cultured murine Melan A cells employing the same umbelliprenin obtained by chemical synthesis as the reference. A parallelism between the content of the coumarin and the recorded depigmenting effect (60% for the EtOH extract of *F. campestris* as the best value) was revealed for all plants extracts when applied at a dose of 100 μg/mL. Our results demonstrate that the same potential of umbelliprenin can be ascribed also to umbelliprenin-enriched plant extracts which reinforces enforce the widespread use of phyto-preparations for cosmetic purposes (e.g., *A. graveolens*).

## 1. Introduction

**Figure 1:**
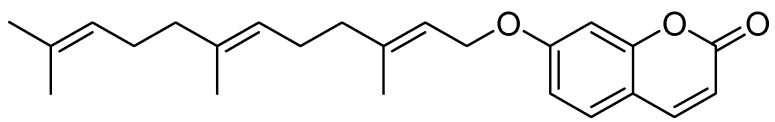
Structure of umbelliprenin (**1**).

During the last two decades extensive studies have been carried on the phytochemical and pharmacological properties of a rare class of secondary metabolites of plant, fungal, and bacterial origin, the oxyprenylated phenylpropanoids and polyketides. In this context umbelliprenin (7-farneseyloxycoumarin, **1**, Figure 1) has been revealed as one of the most promising compounds. *Ferula* [1], *Peucedanum* [2], *Seseli* [3], *Magydaris* [4], *Ammi* [5], *Apium* [6], *Angelica* [6], and *Citrus* [7] species represent the main natural sources of this prenyloxycoumarin.

From a pharmacological point of view, umbelliprenin exhibits anti-inflammatory, immunomodulary, cancer chemopreventive (papilloma, colorectal, melanoma, and breast carcinomas), pro-apoptotic, and anti-melanogenic effects [8,9,10,11]. In particular for this latter biological activity, we have recently shown that umbelliprenin has great potential as a skin whitening agent. Indeed the recorded effects were comparable or even better to those exhibited by known and widely used depigmenting substances like arbutin, kojic acid, some flavonoids, and others [10]. Treatment of skin disorders caused by hyperpigmentation is a topic of current interest. The therapeutic means nowadays at one’s disposal, although very effective, in some cases feature serious side effects. For example arbutin and its aglycone hydroquinone may lead to skin irritation and contact dermatitis [12], while kojic acid is known to be an allergen and to may evoke sensitization [13]. Thus the search for novel, alternative, and more effective agents to modulate skin pigmentation, like umbelliprenin, is a still active and more and more growing field stimulating research. In this short communication, adopting a reverse pharmacognosy approach (e.g., from molecules to plants) [14,15], we wish to describe a simple and reliable HPLC analytical procedure to quantify umbelliprenin in extracts obtained from seeds of selected Apiaceae plant species, namely *Anethum graveolens* L. (dill), *Pimpinella anisum* L. (anise), and *Ferulago campestris* (Besser) Grecescu (field ferula), and to assess the skin whitening properties of the latter using cultured murine Melan A cells as the pharmacological model. Results obtained in our investigation indicate that the HPLC process we set up can be easily adopted to determine the umbelliprenin content of plant extracts in general and that the title species can be effectively considered as therapeutic remedies for the cure of hyperpigmentation syndromes and as ingredients for cosmetic preparations to lighten the skin.

## 2. Results and Discussion

Plant species of the Apiaceae family are nowadays well established to be able to biosynthesize a wide series of oxyprenylated secondary metabolites, in particular umbelliferone derivatives [16]. For our purposes, in this study we selected three species, *A. graveolens*, *P. anisum*, and *F. campestris*, that we have already demonstrated to produce umbelliprenin to a relatively large extent [16,17]. The qualitative and quantitative analysis of this oxyprenylated coumarin was accomplished using four different solvent mixtures, namely EtOH, EtOH/H_2_O 7:3, EtOH/H_2_O 3:7, and a 1.5% solution of β-cyclodextrin (β-CD) in H_2_O, and three different extraction methodologies: “classic” maceration at room temperature (r.t.) for 96 h, ultrasound (US)-assisted and microwave (MW)-assisted processes. US-, MW-, and β-CD-based methods have been chosen on the basis of recent literature data suggesting that both techniques perform satisfactorily in terms of extract yields of a wide panel of biologically active polyphenols and other relevant secondary metabolites [18,19,20]. 

### 2.1. HPLC Analysis

The HPLC qualitative and quantitative analysis of umbelliprenin in the 36 seed extracts obtained from the title plants have been carried out using a Lichrosorb^®^ RP18 column and a mobile phase composed of a mixture of H_2_O, CH_3_CN, and formic acid following the gradient program outlined in the Material and Methods section. Under these experimental conditions the retention time of umbelliprenin was 41.3 (±0.3) min. and we obtained at the same time a complete baseline separation of the analyte without interferences from other detected peaks in all matrices submitted to HPLC analysis. A 13-point calibration curve, obtained at 322 nm using a pure standard obtained by chemical synthesis [17], was plotted using weighted (1/x^2^) linear least-squares regression analysis and was linear over all the concentration range tested r^2^ ≥ 0.9993. The repeatability of the method was assessed by performing six consecutive assays in the same day (within-assay) on quality control (QC) samples spiked at three different standard concentration levels of the pure standard in the range of the calibration curve, namely QC_low_ = 2.5 μg/mL, QC_medium_ = 25 μg/mL, and QC_high_ = 45 μg/mL. QC samples were also examined in separate days to record the between-assay precision (intermediate precision) of the method. The trueness of the set up HPLC methodology was evaluated at the same analyte concentration levels by comparing the measured compound concentrations of the QC samples with their nominal values. All data are reported in Table 1.

The limit of quantification (LOQ) and the limit of detection (LOD), defined according to the ICH International Guidelines “Guidance for Industry on the validation of bio-analytical methods” [21], were 0.5 μg/mL and 0.3 μg/mL, respectively. In all cases the retention time of umbelliprenin in plant extract solution exactly matched that recorded for the pure standard. Finally a total recovery of the analyte > 100% was recorded in all cases.

### 2.2. Quantification of Umbelliprenin in Seeds Extracts

The content of umbelliprenin in extracts of the three title plants obtained by the above mentioned procedures is reported in Table 2. Three independent extractions and analysis to get the final recorded concentrations were accomplished. 

A *t*-test [95% confidence level (ν = 2)] demonstrated that the results for the quantitative determination of umbelliprenin in all plant extracts are not significantly different from the previously cited ones and in all cases experimental *t*-values are lower than the theoretical ones (data not shown). The data reported in Table 2 clearly show that, considering yields, EtOH was the best solvent to accomplish the extraction, with the only exception of *P. anisum* samples, for which a 7:3 EtOH/H_2_O mixture and application of an ultrasounds-assisted procedure led to slightly higher yields. For *A. graveolens* maceration was seen to be the most effective extractive methodology, while ultrasound-based ones provided the best results in the case of both *P. anisum* and *F. campestris*. Prevalence of H_2_O in the extraction medium prevented a full extraction of umbelliprenin from all three seed extracts, even in the presence of an auxiliary agent like β-CD. This latter in other cases was found to efficiently extract apolar compounds structurally related to umbelliprenin [16]. Finally seeds of *A. graveolens* were clearly the richest source of umbelliprenin, with a content nearly 20- to 50-fold higher than the other two Apiaceous species. 

### 2.3. Modulation of Melanogenesis by Seeds Extracts

**Figure 2:**
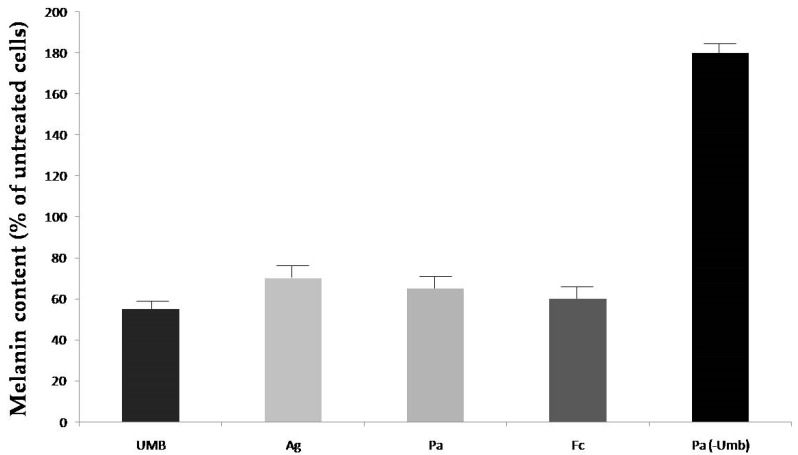
Effect of seeds extracts (100 μg/mL) of *A. graveolens*, *P. anisum,* and *F. campestris* on melanin biosynthesis in Melan-A cells (Umb = Umbelliprenin 40 μM, Ag = *A. graveolens*, Pa = *P. anisum*, Fc = *F. campestris*, Pa-Umb = extract from *P. anisum* with the lowest detected content in umbelliprenin, *p* < 0.05 at Student’s *t*-test).

As a result of the quantification step of our investigation, we selected one extract from each plant, namely those having the highest content in umbelliprenin, for subsequent biological tests and examination of their modulatory properties on melanin biosynthesis using cultured murine Melan A cells as the pharmacological model. Such a pharmacological model proved to be very powerful for our purpose, providing very good and significant results as recently reported for the assay of pure chemically synthesized umbelliprenin [10]. All extracts were assayed at the concentration of 100 μg/mL corresponding to the highest solubility of such products into the medium employed to accomplish biological assays, value at which they did not exert any effect on cell viability. Melanin content was recorded exposing cells to each extract for a period of 48 h. Results are reported in Figure 2. Umbelliprenin, a strong skin whitening agent [16], and a sample of extract with the lowest content of umbelliprenin (e.g., that obtained by extraction with a β-CD aqueous solution and microwave application of seeds of *P. anisum*, see Table 2) were used as references.

We next attempted to rationalize the results in terms of the umbelliprenin concentrations of individual extracts respect to the dose of 100 μg/mL applied, namely 345.7 nM, 14.2 nM, and 60.4 nM for *A. graveolens*, *P. anisum*, and *F. campestris* respectively. From the data reported in Figure 2 it is evident at a glance that the virtual absence of umbelliprenin in the plant extract led to a massive tanning effect (180% respect to untreated control taken as 100%). Values recorded after application of the three plant extract are very similar to each other, ranging from 60% to 70%. Thus it can be hypothesized that the discovery of umbelliprenin-enriched plants with a potential for cosmetic use as depigmenting ingredients and/or umbelliprenin itself could help in the depigmenting properties of plant extracts with other depigmenting phytochemicals. Indeed the activity of *F. campestris* is the most pronounced, despite its lower concentration in this farnesyloxycoumarin. It can be hypothesized that as a component of a phytochemical pool, the action of umbelliprenin as a depigmenting agent may act synergistically and/or antagonistically with other components of plant extracts. To this end it has to be underlined how all seeds of title species are sources of known tanning agents like furanocoumarins, flavonoids like tangeretin and nobiletin, ferulic acid and also of depigmenting ones like quercetin and luteolin [22,23,24,25,26]. Thus the herein observed inhibitory effects of melanin biosynthesis may be the result of the individual contributions of each of the mentioned and other secondary metabolites to the overall modulatory capacity of extracts. To corroborate our hypothesis, the results obtained with the anise seeds extract with the lowest content of umbelliprenin (but reported to have a high content of furanocoumarin [26]) provided a marked tanning activity (180%). In conclusion, in this preliminary investigation we disclose the modulatory properties of seed extracts of selected Apiaceae plant species on melanogenesis. The research on skin pigmenting and whitening agents is a field of remarkable importance for therapeutic and economic aspects, especially when considered in the framework of the cosmetic products market. Profits from the commercialization of skin creams and lotions containing skin tanning or whitening agents have been rapidly increasing in recent years. In the present study we have demonstrated how not only individual phytochemicals, like umbelliprenin, but also enriched plant extracts can be considered as effective skin lightening activators in a non-cancer cell line model. The extracts showed a good cell viability tolerance upon application of doses even > of 100 μg/mL and for prolonged times (48 h, cell viability in the range 92–98%). Dill [27], anise [26], and field ferula [28] derivatives are currently employed as ingredients in skin creams and lotions. Our study reinforces the usefulness of these natural components for cosmetic purposes and provides also further insights into the biological mechanism of action underlying the observed overall skin care effects. It also suggests that other phytopreparations (e.g., alcoholic extracts, apolar fractions, etc.), other than the widely used essential oils obtained by steam distillation from the title plant seeds, could be added as ingredients of cosmetic skin formulations. The present communication also provides a validated analytical HPLC protocol that can be easily adopted and used for identiification, quality control, and determination of the chemical fingerprint profiles of umbelliprenin-containing plant extracts. 

## 3. Materials and Methods 

### 3.1. Chemicals and Reagents

Umbelliferone, all *trans* farnesyl bromide and β-cyclodextrin, (purity >95% as declared by the suppliers), were purchased from Merck Sigma-Aldrich (St. Louis, MO, USA). Umbelliprenin was also synthesized following the recently reported method and its purity (>98.6%) assessed by GC/MS and ^1^H-NMR [10]. Formic acid and acetonitrile (HPLC-grade) were purchased from Dasit Carlo Erba (Milan, Italy) and used without further purification. Double-distilled water was obtained from a Millipore Milli-QPlus Waters treatment system (Millipore Bedford Corp., Bedford, MA, USA).

### 3.2. Plant Samples and Extraction

Seeds of *A. graveolens* L., *P. anisum* L., and *F. campestris* have been purchased from a local market in Pescara (Abruzzo Region, Italy). All samples were identified by the Italian authors. Voucher specimens named AG-S-02, PA-S-02, and FC-S-01, respectively, have been deposited at the laboratory of Chemistry of Natural Compounds at the Department of Pharmacy of the University “G. D’Annunzio” of Chieti-Pescara. All samples have been ground and homogenized prior to extraction experiments. The extractive procedure followed was the same as recently described in the literature [16]. Each solid extract was suspended in 100 μL of MeOH, filtered and an aliquot of 20 μL of the resulting solution injected into the HPLC apparatus. 

### 3.3. HPLC 

HPLC analyses were accomplished using a Waters liquid chromatograph system (Waters, Milford, MA, USA) equipped with a model 600 solvent pump and a 2996 photodiode array detector. Empower v.2 Software (Waters) was used for data acquisition. Chromatography of each extract sample was performed employing a C18 reversed-phase packing column (LichroSorb RP18, 4.6 × 150 mm, 5 μm, Merck, Darmstadt, Germany). The column was thermostatated at 25 °C ± 1 °C using a Jetstream2 Pluscolumn oven. The UV/Vis acquisition wavelength was set in the range of 210–600 nm. Quantitative analyses were determined with a selective detection set at 322 nm. Gradient elution mode adopted was the same as already recently reported [16]. The mobile phase was degassed using a Degassex apparatus mod. DG-4400 (Phenomenex, Torrance, CA, USA). Relevant parameters like calibration, linearity, LOD, and LOQ have been calculated following our already described procedure [16].

### 3.4. Cell Culture

Melan-a cells, an immortalized mouse melanocyte cell line, were obtained from the Wellcome Trust Functional Genomics Cell Bank (London, UK). Cells were maintained in RPMI 1640 (Lonza, Basel, Switzerland) supplemented with 10% fetal bovine serum, 50 U/mL penicillin, 50 U/mL streptomycin (PS Lonza) and 200 nM PMA (phorbol 12-myristate 13-acetate; Sigma). Cells were incubated at 37 °C in a humidified 5% CO_2_/air atmosphere. The stock solution of umbelliprenin and plant extracts were prepared in DMSO (1000×) and were stored at −20 °C until use. Work solutions were freshly prepared for each experiment with a final DMSO concentration of 0.1% and with concentrations as described in previous paragraphs. Controls were always treated with the same amount of DMSO (0.1%, *v*/*v*) as used in the corresponding experiments. 

### 3.5. Cell Viability Assay

Non-tumoral murine melanocytes were seeded at 60.000 cells on six plate wells and treated for 48 h with umbelliprenin or plant extracts at the indicated concentrations or DMSO. Cells were detached by trypsinization, collected in phosphate buffer saline and centrifuged at 1500 rpm for 5 min at 4 °C. Cells pellets were re-suspended in the trypan blue solution (0.25%, *w*/*v* in PBS) and counted in a Malassez cell under a light microscope. The percentage of cell viability was calculated using the following formula: % cell viability = [1 − (blue cells/total cells)] × 100.

### 3.6. Melanin Content Measurement

Melan-a-cells. non-tumoral murine melanocytes were seeded at 60,000 cells on six plate wells and treated for 48 h with the indicated doses of umbelliprenin and plant extracts or carrier solvent (DMSO). 5 × 10^6^ cells were centrifuged at 1500 rpm for 5 min at 4 °C. The cell pellet was washed twice with phosphate buffer saline, transferred in an Eppendorf vial and centrifuged at 5000× *g* for 5 min at 4 °C. The supernatant was discarded. Two hundred μL of water and 1 mL of EtOH/diethyl ether (1/1) were added to remove opaque substances other than melanin. The mixture was incubated for 15 min. at r. t., centrifuged at 5000× *g* for 5 min. and the supernatant was discarded. The precipitate containing melanin was solubilized by 300 μL of a mixture of NaOH (aq) 1 M/DMSO 9: 1 after heating at 80 °C for 1 h. The absorbance was measured at 405 nm. The melanin content was expressed as a percentage of control (=100%). UV experiments have been performed following the method by Liebermann and Hopkins using a UVX radiometer (UVP, Inc., Upland, CA, USA) [29].

## 4. Patents

(1) DeMedina: P.; Bize, C.; Rives, A.; Paillasse, M.; Epifano, F.; Genovese, S. Depigmenting agents to lighten the skin, EP15305836.7; (2) DeMedina, P.; Bize, C.; Rives, A.; Paillasse, M.; Epifano, F.; Genovese, S. Skin pigmentation modifiers to darken or lighten the skin, WO 2016/193220 A1.

## Figures and Tables

**Table 1:** Within-assay and between-assay precision (RSD%) and trueness (bias%) of the HPLC run.

	QC_low_	QC_medium_	QC_high_
**Within assay**			
Mean back-calculated	2.49	24.98	44.96
RSD%	3.48	4.02	3.09
Bias%	−0.12	−0.48	−1.96
**Between assay**			
Mean back-calculated	2.49	24.91	44.92
RSD%	4.47	3.97	3.06
Bias%	−0.29	−0.22	−0.33

**Table 2:** Quantification of umbelliprenin in seed extracts of *A. graveolens*, *P. anisum,* and *F. campestris* (Extracts used in the subsequent tests are marked in bold).

	A *	B *	C *	D *
*A. graveolens*				
1 **	1267.77 ± 7.43	22 ± 1.24	ND	0.6 ± 0.05
2 **	544.21 ± 3.11	14.22 ± 0.95	ND	ND
3 **	122.44 ± 2.98	11.33± 0.91	ND	ND
*P. anisum*				
1 **	43.12 ± 1.77	24.15 ± 0.87	1.16 ± 0.04	7.08 ± 0.54
2 **	35.76 ± 1.11	52.32 ± 2.12	0.89 ± 0.05	9.54 ± 0.61
3 **	38.44 ± 1.16	7.15 ± 0.44	1.02 ± 0.04	0.44 ± 0.02
*F. campestris*				
1 **	112.66 ± 3.91	8.16 ± 0.26	ND	ND
2 **	221.35 ± 1.06	15.4 ± 0.81	ND	ND
3 **	133.48 ± 2.43	9.14 ± 0.53	ND	ND

* Values expressed as μg/g dry extracts, A = EtOH, B = EtOH/H_2_O 7:3, C = EtOH/H_2_O 3:7, D = aqueous 1.5%β-CD; ** 1 = maceration (96 h), 2 = Ultrasounds, 3 = Microwaves, ND = Not Detected (below LOD).
